# AnnoMe: user-defined classification of HR-MS/MS spectra for natural product discovery

**DOI:** 10.1093/bioadv/vbag111

**Published:** 2026-05-21

**Authors:** Christoph Bueschl, Tomas Rypar, Lenka Molcanova, Juraj Markus, Bernhard Seidl, Maria Doppler, David Ruso, Christina Maisl, Karel Smejkal, Rainer Schuhmacher

**Affiliations:** Department of Agricultural Sciences, Institute of Bioanalytics and Agro-Metabolomics (iBAM), BOKU University, Vienna, 1180, Austria; Department of Agricultural Sciences, Institute of Bioanalytics and Agro-Metabolomics (iBAM), BOKU University, Vienna, 1180, Austria; Department of Chemistry and Biochemistry, Mendel University in Brno, Brno, 613 00, Czech Republic; Department of Natural Drugs, Faculty of Pharmacy, Masaryk University, Brno, 612 00, Czech Republic; Department of Natural Drugs, Faculty of Pharmacy, Masaryk University, Brno, 612 00, Czech Republic; Core Facility Bioactive Molecules: Screening and Analysis, BOKU University, Tulln, 3400, Austria; Core Facility Bioactive Molecules: Screening and Analysis, BOKU University, Tulln, 3400, Austria; Core Facility Bioactive Molecules: Screening and Analysis, BOKU University, Tulln, 3400, Austria; Department of Agricultural Sciences, Institute of Bioanalytics and Agro-Metabolomics (iBAM), BOKU University, Vienna, 1180, Austria; Department of Natural Drugs, Faculty of Pharmacy, Masaryk University, Brno, 612 00, Czech Republic; Department of Agricultural Sciences, Institute of Bioanalytics and Agro-Metabolomics (iBAM), BOKU University, Vienna, 1180, Austria

## Abstract

**Summary:**

Annotation of HR-MS/MS spectra is a complex task that can be tackled either by expert interpretation or machine learning models that rely on large spectral/structural databases for training. Frequently, users want to find novel compounds of a particular substance class they are already familiar with. This requires the classification of detected compounds as “relevant” (i.e. belonging to the compound class of interest) or not (i.e. “other”). For such applications, the python-based AnnoMe software is presented that allows users to classify their experimental HR-MS/MS spectra according to their aims. By leveraging a user-curated dataset of “relevant” and “other” reference HR-MS/MS spectra alongside structure-informed embeddings (MS2DeepScore), the package enables rapid and accurate prediction of “relevant” compounds with custom-trained classification models and a majority vote, facilitating exploration of the complex chemical space inherent to LC-HRMS/MS data. This software is demonstrated by predicting putative prenylated flavonoids for prioritization in natural product discovery.

**Availability and implementation:**

Code, documentation, and datasets are available at https://github.com/chrboku/AnnoMe and https://zenodo.org/records/16322488.

## 1 Introduction

Natural product discovery strategies rely on prioritization of candidate molecules from liquid chromatography high resolution tandem mass spectrometry (LC-HR-MS/MS) datasets. Researchers often seek “more of the same,” e.g. new metabolites of a particular chemical class, because structural similarity often correlates with bioactivity, mechanistic hypotheses, and downstream validation feasibility (Wolfender *et al.* 2019). However, screening for class-consistent candidates at scale is challenging as spectral libraries are incomplete (Hoffmann *et al.* 2022) and manual, rule-based searches are labor-intensive. Consequently, there is a need for flexible tools that help accelerate semi-targeted dataset exploration. In addition, as resolving the complete chemical structure of novel compounds is laborious and generally requires orthogonal analytical techniques like NMR spectroscopy, the prioritization of the detected metabolites is of utmost importance in natural product discovery (Kim *et al.* 2010).

To tackle this challenge, machine learning (ML) has developed into an essential tool in bioinformatics and is also rapidly transforming the field of analytical chemistry, particularly for tackling the complexity of LC-HR-MS/MS data. Using analytical characteristics such as mass-to-charge ratio (*m/z*), HR-MS/MS fragmentation spectra, and retention time, ML enables to predict and interpret compound or sample classifications, and also facilitates structure elucidation where traditional methods are limited by manual annotation challenges (Kim *et al.* 2010, Hong *et al.* 2025, Liu *et al.* 2025).

ML approaches have shown potential to overcome key challenges in compound annotation via *in silico* HR-MS/MS spectra predictions (Wang *et al.* 2021, Murphy *et al.* 2023), structure prediction from fragmentation spectra (Dührkop *et al.* 2019, Stravs *et al.* 2022) to find motifs (i.e. HR-MS/MS fragment subsets) of structural features/substructures (Ortega *et al.* 2025), or improving spectral matching through automated pattern extraction (Huber *et al.* 2021, Guo *et al.* 2023, Bushuiev *et al.* 2026). MS2DeepScore has achieved notable success in clustering large spectral datasets and improving library matching by learning structure-informed spectral embeddings from fragmentation patterns (Huber *et al.* 2021).

Numerous supervised classifiers have been reported for diverse tasks such as predictions of chemical class (Picache *et al.* 2020), or even antimicrobial resistance directly (Weis *et al.* 2022). Others can predict bioactive scaffolds using molecular fingerprints (Brittin *et al.* 2025), or find bioactive compounds (Brown *et al.* 2022).

In this study, we present the python-based software AnnoMe for supervised classification of HR-MS/MS spectra. By leveraging a user-curated dataset consisting of a set of diverse reference spectra (i.e. derived from “relevant” and “other” compounds) alongside MS2DeepScore embeddings, it enables accurate prediction of novel compounds, facilitating exploration of the complex chemical space according to the chemical class of interest to the user. Among others, explorative studies can aim at prioritizing (i) novel terpenoids, (ii) phenylpropanoids, or (iii) indole alkaloids. Here, an example of the workflow is presented with the search for novel prenylated flavonoid metabolites in *Paulownia tomentosa* fruit extracts.

## 2 Workflow overview and methods

**Figure 1:**
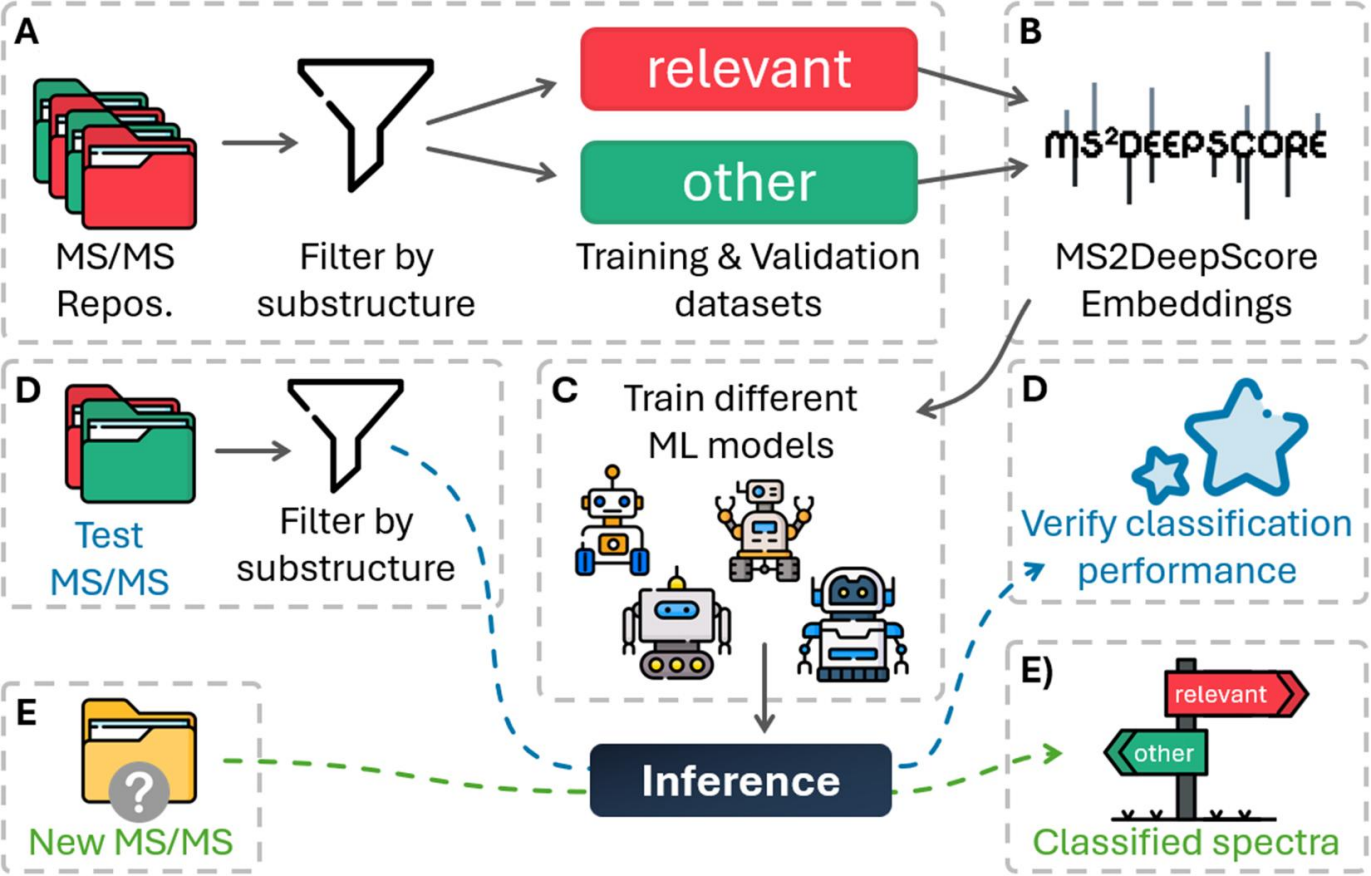
Overview of the workflow. (A) Reference HR-MS/MS datasets of “relevant” and “other” compounds are established. (B) Structural-embeddings are calculated via MS2DeepScore. (C) A series of machine learning models is trained to differentiate between the two classes. (D) An optional test dataset is used to assess the model’s performance. (E) HR-MS/MS spectra of unknowns are classified as “relevant” or “other.”

The AnnoMe package is designed to assist the user in prioritizing metabolic features (i.e. HR-MS/MS spectra) by assigning them to one of two orthogonal classes. The first class “relevant” represents compounds the user is interested in, while compounds the user is not interested in are assigned to the “other” class. What classifies as “relevant” is to be defined by the user and can be, e.g. the presence of a particular substructure. After the ML models have been trained, novel spectra of unknown compounds can also be classified as either “relevant” or “other” guiding further exploration of the dataset toward the user’s “relevant” compounds. The individual steps of the workflow (Fig. 1) are:

- The user provides sets of HRMS/MS spectra representing the two classes “relevant” and “other” (MGF files). These spectra can be obtained by (i) filtering public spectral repositories, or (ii) be compiled from in-house spectral libraries. For substructure-based filtering, the AnnoMe package provides functions and a graphical user interface to filter based on SMARTS strings (https://daylight.com/dayhtml/doc/theory/theory.smarts.html).
- Once the training and testing datasets have been established, MS2Deepscore embeddings are calculated for each HR-MS/MS spectrum. Instead of relying solely on peak intensity overlaps (classical cosine similarity), MS2DeepScore learns to map HR-MS/MS spectra into a 500-dimensional numerical representation (embedding) that reflects the underlying chemical structure. For more details the reader is referred to Huber et al. (2021).
- In the next step, different machine learning models (e.g. LDA, SVM, NeuralNet; Supplementary Information SI1, available as supplementary data at *Bioinformatics Advances* online) are trained on the calculated embeddings, to optimize for correct prediction of the classes based on the training data. Cross-fold validation (the actual strategy is to be defined by the user, stratified CV per default) is carried out and different performance metrics (balanced accuracy, F1 score, confusion matrices) alongside diagnostic plots are automatically generated for final review.
- The user can optionally specify a test dataset consisting of HR-MS/MS spectra of known “relevant” and “other” compounds to test the model’s performance on data not used during training.
- Finally, unclassified, experimental spectra (e.g. from an explorative study) are also classified utilizing the trained model.

The AnnoMe package is implemented in the Python programming language. It utilizes MS2DeepScore for structural embeddings, and the scikit-learn package for classification tasks (Huber *et al.* 2021). It offers modules for downloading common, public HR-MS/MS libraries (e.g. MoNA, MassSpecGym, MSnLib), parsing and overview generation (polarity, fragmentation energies, etc.), and substructure-based filtering via SMARTS strings. The user has the possibility to set hyperparameter values for the classifiers, the cross-validation strategy, and configure the majority vote to avoid overfitting. The software can be used programmatically (e.g. in a Jupyter notebook) or interactively (i.e. graphical user interfaces for (i) substructure-based filtering and (ii) classification training and inference), with the latter also conveniently visualizing matching and non-matching structures for SMARTS string filters and the classifier’s performance (F1, accuracy). The software does not require high performance computing resources and has been tested successfully on a standard laptop (Intel Core Ultra 5 125U, 12 cores; 16GB memory; SSD; Win11) with 655 000 loaded HR-MS/MS spectra. The package, documentation, and a comprehensive tutorial (exemplified with prenylated flavonoids) are available at https://github.com/chrboku/AnnoMe/releases/tag/v1.0.0.

## 3 Results and discussion

### 3.1 Demonstration of (iso-)flavonoid prediction trained on public repository data

The developed AnnoMe package was tasked with classifying (iso-)flavonoids as an example. For this, the MSnLib (Brungs 2025, https://zenodo.org/records/15683662, version v6) dataset was filtered with respective SMART strings (Supplementary Information SI2, available as supplementary data at *Bioinformatics Advances* online), resulting in 655 (iso-)flavonoids (class “relevant”), while 10 000 non-(iso-)flavonoids were used as the “other” class. Furthermore, another 192 (iso-)flavonoid and 249 “other” compounds were measured in-house and used to test the classifier’s performance. Of these, 74 (iso-)flavonoids were unique to the test dataset. For details of the LC-HRMS/MS measurements, please refer to Supplementary Information SI3-5, available as supplementary data at *Bioinformatics Advances* online. Separate classifiers were trained for ionization polarity (pos/neg) and collision energy settings (20 eV, 30 eV, 60 eV, stepped collision energy [20, 45, 70 eV]). After training, the test datasets showed average balanced accuracy rates of 0.866 (sd 3%, min 0.818, max 0.908) for the training set and 0.82 (sd 4%, min 0.76, max 0.87) for the test set across the spectral subsets, with true-positive rates above 55% and 82% on average, while the false-positive rates were below 10% and 5.2% on average, meaning that the classifier did not incorrectly predict a truly “other” compound as a “relevant” one (Table ST1, available as supplementary data at *Bioinformatics Advances* online), suggesting that the classifier successfully learned to differentiate between (iso-)flavonoid and other compounds.

### 3.2 Demonstration of prenylated flavonoid prediction trained on public repository data

AnnoMe’s performance is further demonstrated for prenylated flavonoids, which are bioactive metabolites, produced by plants such as *Paulownia tomentosa* trees (Cheng *et al.* 2019). The MSnLib was filtered for prenylated flavonoids (class “relevant”), while structures not matching any of the filters were assigned to the class “other.” Furthermore, an independent test set consisting of HR-MS/MS spectra obtained from 64 prenylated flavonoids (class “relevant”) and HR-MS/MS spectra of a wheat ear extract (class “other”; wheat does not produce prenylated flavonoids) was compiled and used to test the performance of the trained ML models (Supplementary Information SI3-5, available as supplementary data at *Bioinformatics Advances* online). A minor subset of 17 prenylated flavonoids was present in both libraries, while 174 and 47 prenylated flavonoids were unique for the MSnLib or the in-house dataset respectively, making the test set largely distinct from the training set with respect to the “relevant” compounds (Supplementary Information SI6, available as supplementary data at *Bioinformatics Advances* online, shows different plots of the complementarity of the training and test sets as well as dimensionality reduction plots). The resulting training and test sets consisted of 30 703 and 7312 spectra, respectively. Training was done separately for each ionization polarity. With the graphical user interfaces, most steps (e.g. compound and meta-information filtering) completed in a couple of minutes, while the main steps (calculation of embeddings and training) took 3:45 hours and 40 minutes respectively on a standard laptop (Intel Core Ultra 5 125U, 12 cores; 16GB memory; SSD; Win11).

Obtained model performance metrics are shown in Table 1. In the training set, the models showed both high sensitivity and specificity, while in the validation set, these values were reduced. Moreover, a test consisting of 155 non-prenylated (iso-)flavonoid compounds from the in-house library served as a challenging ground-truth negative control (Table ST2, available as supplementary data at *Bioinformatics Advances* online). Here, with the trained classifiers 58% and 67% (negative and positive modes respectively) of the HR-MS/MS spectra were correctly assigned to non-prenylated compounds (“other” class). In summary, the trained models successfully predicted the classes correctly for most of the tested compounds but struggled with structurally similar compounds.

**Table 1:** Prediction performance for the prenylated flavonoids trained on the MSnLib.^a^

Split	pol.	n_rel.	n_other	TP	FN	TN	FP	bal.acc.
Train	neg	71	10 095	100.0%	0.0%	97.5%	2.5%	0.9874
	pos	28	20 509	100.0%	0.0%	98.4%	1.6%	0.992
Validation	neg	115	3154	76.5%	23.5%	83.5%	16.5%	0.8002
	pos	115	3928	77.4%	22.6%	94.7%	5.3%	0.8606

a pol: polarity, n_rel: number of “relevant,” n_other: number of “other,” TP: true-positive, FN: false-negative, TN: true-negative, FP: false-positive, bal.acc: balanced accuracy.

A comparison with SIRIUS (Dührkop *et al.* 2019, version 6.2.2, parameters are listed in Supplementary Information SI7, available as supplementary data at *Bioinformatics Advances* online) on the test dataset showed that more of the prenylated flavonoids (ground truth) were classified correctly with the AnnoMe-established prediction models (average 77% in the two test sets, Table ST2, available as supplementary data at *Bioinformatics Advances* online), which is higher than that obtained with SIRIUS (41% of the spectra were predicted as prenylated flavonoids within the ClassyFire ontology, category “most specific class”). The results demonstrate that the trained classifier is suited to predict compounds belonging to the class of prenylated flavonoids, when trained on publicly available reference datasets only. However, it should also be noted that SIRIUS predicts different compound classes simultaneously and is therefore more versatile for comprehensive metabolomics dataset annotation, while AnnoMe can be used as a complementary tool for data exploration and compound prioritization.

### 3.3 Demonstration with in-house prenylated flavonoids

Similar to using HR-MS/MS spectra from public repositories, classifiers can also be trained on in-house libraries (e.g. different fragmentation method, particular substance class of interest that is under-represented in public libraries). However, as in-house libraries are typically smaller in size than public repositories, care needs to be exercised to make sure that the class “other” is adequately covered.

For this demonstration, an in-house library consisting of HR-MS/MS spectra derived from some 417 natural products, mainly from plants and fungi, plus 64 prenylated flavonoids were used. Additionally, HR-MS/MS spectra obtained from specialized metabolites of a flowering wheat ear were used to enrich the dataset with the class “other.”

Cross-validation showed that the specificity (min 99.4%) was higher than the sensitivity (average 75.7%, min 56.4%, max 87.7%), suggesting that almost all the spectra of the class “other” were correctly predicted (Table ST3, available as supplementary data at *Bioinformatics Advances* online). Different collision energy setups performed better or worse, thus it is recommended to not merge different collision energy setups for the classification task.

The trained classifier was then utilized to predict prenylated flavonoids in *Glycyrrhizza uralensis* root and *Paulownia tomentosa* fruit extracts (Table ST4, available as supplementary data at *Bioinformatics Advances* online). For the two ionization polarities, 231 and 305 HR-MS/MS spectra (25% and 15%) were predicted to be prenylated flavonoids, while for *Paulownia tomentosa* between 171 and 1005 HR-MS/MS spectra (17% to 34%) were predicted to be prenylated flavonoids in the different samples, some of which were subject to be studied further (Rypar *et al.* 2025).

### 3.4 Performance with respect to the number of spectra used for training of the classifier

To estimate the number of HR-MS/MS spectra necessary to achieve high classification performance, the data from public repository training (Section 3.2) was reused and the number of contained spectra randomly reduced in 10% steps. Comparable performance values (on average 62% of the “relevant” class were correctly predicted) were observed for the first five reduction steps (i.e. 100% down to 50% of the HR-MS/MS spectra used as “relevant”), indicating that for the tested dataset, the classifier needed at least some 1000 HR-MS/MS spectra of the “relevant” class to differentiate them from HR-MS/MS spectra of the class “other.” Further details of this investigation are available in Supplementary Information SI8, available as supplementary data at *Bioinformatics Advances* online. It should be noted that the number of 1000 spectra required for highly accurate classification of prenylated flavonoids may not generalize to other substance classes. Thus, users are encouraged to determine the required number of spectra for their specific applications.

## 4 Conclusion

Many untargeted metabolomics studies are guided by certain chemical structures known to be relevant (i.e. from literature or previous experiments). However, finding such novel compounds is difficult with HR-MS/MS spectra only. The developed AnnoMe software for HR-MS/MS spectra classification offers a practical framework for the metabolomics community. By enabling users to easily filter public datasets, custom-train prediction models, and classify spectra as “relevant” or “other” with high accuracy, the AnnoMe package addresses a common challenge in metabolomics. Validation results demonstrate an average balanced accuracy of 0.84 in correctly predicting compound classes while having a high sensitivity, providing users with confidence in the predictions.

By utilizing public HR-MS/MS spectral repositories, which are nowadays easily accessible, a wealth of data for training is available and SMART codes allow for customizable, fast sampling of relevant structures for training. This significantly improves the classification of compounds and highlights the importance of open data sharing. As many untargeted metabolomics studies are guided by certain chemical structures known to be relevant from literature or previous experiments, annotating such novel compounds is critical.

## Supplementary Material

vbag111_Supplementary_Data

## Data Availability

Supplementary data and HR-MS/MS spectra of standards, *Paulownia tomentosa*, and *Glycyrrhizza uralensis* extracts are available at *Bioinformatics Advances* online and on Zenodo (https://dx.doi.org/10.5281/zenodo.16322488). Raw-data files of the LC-HRMS measurements of the *Paulownia tomentosa* extracts are available on GNPS/MassIVE (https://massive.ucsd.edu) under the ID MSV000098757.
