# Supplementary material for: AnnoMe: user-defined classification of HR-MS/MS spectra for natural product discovery
Source: Bioinform Adv. 2026 May 21;6(1):vbag111. doi: 10.1093/bioadv/vbag111 (PMC13192349; doi:10.1093/bioadv/vbag111)
Supplement: vbag111_Supplementary_Data [file vbag111_supplementary_data.zip › SupplementaryInformation.pdf]

Supplementary Information for

AnnoMe: User-defined classification of HR-MS/MS spectra for natural product discovery

Bueschl Christoph[1*], Rypar Tomas[1,2], Molcanova Lenka[3], Markus Juraj[3], Seidl Bernhard[4], Doppler Maria[4], Ruso David[4], Maisl Christina[1], Smejkal Karel[3], Schuhmacher Rainer[1]

[1]BOKU University, Institute of Bioanalytics and Agro-Metabolomics (iBAM), Department of Agricultural Sciences, Vienna, Austria, [2]Mendel University in Brno, Department of Chemistry and Biochemistry, Brno, Czech Republic, [3]Department of Natural Drugs, Faculty of Pharmacy, Masaryk University, Brno, Czech Republic, [4]BOKU University, Core Facility Bioactive Molecules: Screening and Analysis, Tulln, Austria

Contents

Supplementary Information S1 2
Supplementary Information S2 3
Supplementary Information S3 4
Supplementary Information S4 5
Supplementary Information S5 7
Supplementary Information S6 8
Supplementary Information S7 10
Supplementary Information S8 11

Supplementary Information S1
Machine Learning Models available in AnnoMe

- K Nearest Neighbors
- Linear SVM
- Decision Tree
- Random Forest
- Neural Net
- AdaBoost
- Naïve Bayes
- QDA
- LDA
- Extra Trees
- Bagging Classifier
- Logistic Regression
- Ridge Classifier
- Voting Classifier (soft)
- Furthermore, any classification algorithm of the scikit-learn package can be used (https://scikit-learn.org/stable/supervised_learning.html).

Supplementary Information S2
Overview of SMART Strings used for finding Prenylated (Iso-)Flavonoids

Overview of SMART strings and their illustrations using the SMART.plus portal (https://smarts.plus/).

Description	SMARTS string<br>(underlined ones are illustrated)	Illustration
Flavonoid core	<u>O=C@1@C@C(@O@C2@C@C<br>@C@C@C@1@2)C@3@C@C@<br>C@C@C3</u>	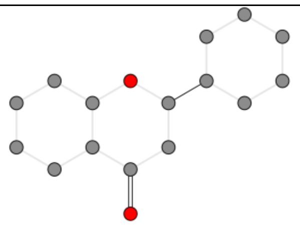
Isoflavonoid core	<u>O=C@1@C@2@C@C@C@C@C<br>@2@O@C@C@1C@3@C@C@C<br>@C@C@3</u>	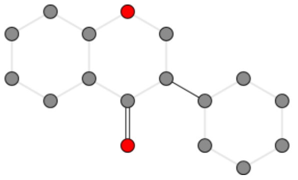
Various prenyl groups	<u>CC=C(C)CCC=C(C)C</u><br>C\\C=C(/C)\\CCC(O)C(=C)C<br>CC(O)C(C)CCC=C(C)C<br>CC=C(C)CCCC(C)(C)O<br>CC(=CCCC1(C)OCCC=C1)C<br><u>CC(=C)C(O)CCC1(C)OC=CCC1O</u><br>C\\C=C(/C)\\CCCC(C)(O)CO<br>CC(=CCCC(C)(O)C1CCCO1)C<br>CC1(C)C(O)CCC2(C)OCCCC12<br>CC1C(=CCC(O)C1(C)C)C<br><u>CC1C(=C)CCC(O)C1(C)C</u><br>COCC(C)(O)CCCC(=CC)C<br>CC(=CCCC1(C)OC=CCC1O)C<br>CC1(C)OCCC=C1<br>CC1OC=CC1(C)C<br>CC(CC=C(C)C)C(=C)C<br>CCC(=C)C<br>CC=C(C)C<br><u>C-C(-C)-C=C</u><br>CC(=C)C=C<br>CC(=CCO)C<br>CC(C)(O)C=C<br>CCC(C)(C)O<br><u>CC(O)C(=C)C</u><br>CC1OC1(C)C<br>C\\C=C(/C)\\CO<br><u>CC(O)C(C)(C)O</u><br>CC1(C)CCc2ccccc2O1<br><u>CC1(C)CCCCO1</u>	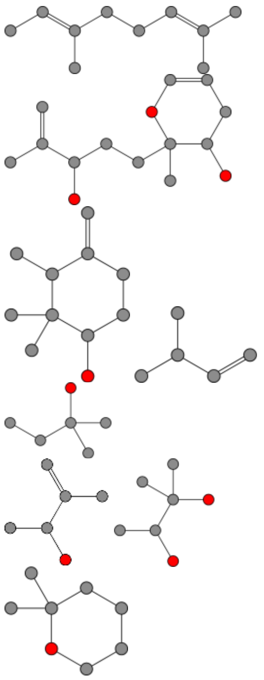

Supplementary Information S3
LC-HRMS measurements of prenylated (iso-)flavonoids

First, stock solutions were prepared from previously isolated, prenylated compounds and reference standards of polyphenols (mainly flavonoids) by dissolving 1 mg of the pure substance in 1 mL of MeOH or 1/1 mix of MeOH/DMSO (v/v) if poor solubility was observed. The stock solutions were further diluted by MeOH to obtain a concentration of 1 µg/mL for the measurement solutions.

Then, the measurement solutions were analyzed by LC-HRMS/MS under standardized conditions using 20 minutes long linear gradient method (MeOH and water both acidified with 0.1% formic acid (v/v)) on a C18 reversed-phase chromatographic column (3.5 µm, 2.1 mm × 150 mm, XBridge® Waters, Milford, MA, USA). For further information refer to https://link.springer.com/article/10.1007/s00216-022-04134-z. 2 µL of the solutions were injected and full scan mass spectra in fast polarity switching mode were collected to evaluate suitable adducts for targeted HRMS/MS data acquisition in subsequent injections. At least two adducts were recorded for each compound, which most commonly were protonated and deprotonated, in positive and negative ionization modes ($[M+H]^+$ and $[M-H]^-$), respectively. Moreover, the abundances of the selected adducts were evaluated and the injection volume in the subsequent MS/MS data acquisition measurements was adapted to reach intensity levels of 1E7 of the precursor ion in consecutive scans. Separate injections for each adduct were used to acquire fragmentation spectra with different collision energies (HCD: '10', '20', '30', '40', '50', '60', '70', '80', '90', '100' and 'stepped 20, 45, 70 eV' and CID: '10', '20', '30', '35', '40', '50', '60', '70', '80', '90', '100 eV' in absolute values) using targeted HRMS/MS acquisition (tMS2 mode) with a specified RT window and precursor mass. At least 5 fragmentation spectra for each adduct and collision energy were required for further processing.

Collected raw LC-HRMS/MS data were processed by an adopted, automatic workflow using the RMassBank package (Stravs, MA.; Schymanski, EL.; Singer, HP.; Hollender, J., Automatic recalibration and processing of tandem mass spectra using formula annotation. *Journal of Mass Spectrometry* **2013,** *48* (1), 89-99.).

Supplementary Information S4
LC-HRMS measurements of plant extracts

Immature fruits of *Paulownia tomentosa* were collected and extracted with ethanol approximately 1:1 (w:v) three times to yield ethanolic extracts. Then liquid-liquid extraction was performed. Briefly, a part of the ethanolic extract was dissolved in 90% methanol and extracted with hexane (four times) to obtain the hexane portion. Methanol was evaporated and the rest was diluted with water and extracted with chloroform (4x) to obtain the chloroform portion. The water part was subsequently extracted with ethyl acetate (4x) to obtain the ethyl acetate portion and the water portion.

For fruits collected in October 2022 (PT22), the chloroform portion was further separated by flash chromatography on silica packed columns yielding ten fractions, from which fraction PT22-CH/5-6, PT22-CH/7-11, PT22-CH/15-18 and whole chloroform portion PT22-CH were further used and analyzed. All fractions were dried under nitrogen flow, under vacuum, lyophilized and stored at 8°C.

For fruits collected in October 2024 (PT24), the chloroform portion was further separated by column chromatography on silica gel with methanol:toluene:chloroform (3/5/92, v/v/v) mobile phase composition. With gradually increased polarity of the mobile phase, a total of 109 fractions were collected (125 mL each). These were separated and analyzed with thin layer chromatography (TLC) and fractions with similar profiles were merged, which resulted into 16 fractions. Of these, the fractions PT24-CH/24-26, PT24-CH/27-28, PT24-CH/51-53 and the whole chloroform portion PT24-CH were further analyzed. All fractions were dried under nitrogen flow, under vacuum, lyophilized and stored at 8°C.

Glycyrrhiza uralensis (GU) plant material was obtained in collaboration with the Faculty of Horticulture, Mendel University. Roots and rhizomes were harvested in September 2024. The material (2.2 kg) was mechanically processed by crushing it with a hammer and cutting it into small pieces using an axe. Extraction was performed four times using 4 L of ethanol and sonication. The combined extracts were concentrated under reduced pressure, and residual water was removed by freeze-drying to yield the crude extract, which was stored at 8°C.

The wheat ear sample was prepared as follows: wheat ears were harvested after 96h post flowering stage by cutting the entire ear and immediately freezing it in liquid nitrogen. Then, the samples were stored at -80°C until further processing. For extraction, 100 ± 2 mg of frozen, milled plant material were thawed in 0.5 mL MeOH/ACN/H_2O 3/3/2 (v/v/v) + 0.1 % FA (three volumes of methanol were mixed with three volumes of acetonitrile and two volume of water, both acidified with formic acid to a concentration of 0.1 %, v/v), vortexed and kept in an ultrasonic bath for 15 min. After centrifugation (14,000 rpm, 10 min), the supernatant was diluted with 0.1 % aqueous FA (v/v) to a final ratio of MeOH/ACN/H_2O 1/1/2 (v/v/v) + 0.1 % FA and centrifuged again.

For the LC-HRMS/MS measurements, 0.25 mg/mL solutions in MeOH were prepared for PTCH-22 and PTCH-24 samples and 0.5 mg/mL solution for GU extract. The wheat sample was used without further processing. All samples were injected in a volume of 2 µL and analyzed by high-performance liquid chromatography coupled to high-resolution mass spectrometry (LC-HRMS). An UHPLC Vanquish system (Thermo Scientific, Bremen, Germany) was used for chromatographic separation with a versatile 47 minutes long reversed-phase chromatography method using 100 mm × 2.1 mm, 2.7 µm analytical HPLC column Ascentis Express RP-Amide (Sigma-Aldrich, USA) and linear gradient elution with ACN (B) and water (A) both acidified with FA (0.1%, v/v) were used. The gradient ran from 10% to 100% B over 36 minutes, followed by a 4-minute hold and re-equilibration at 10% from 40.1 to 47 minutes. The flow-rate was set to 0.3 mL/min. The column was kept at 40°C and the autosampler at 10°C during the analysis. An Orbitrap IQ-X (Thermo Scientific, Bremen, Germany) equipped with heated electrospray ionization source was utilized for data acquisition using the AcquireX workflow. Briefly, mass spectra were recorded in profile full scan mode with resolving power settings 120,000 at *m/z* 200 in negative or positive ionization modes for generation of exclusion list from blank and inclusion lists of detected features from the samples. One full scan and two data-dependent fragmentation spectra using HCD 40 eV and stepped HCD (20, 45, 70 eV) were utilized. The fragmentation mass spectra were recorded using the data-dependent mode first acquiring profile full scan spectra with a resolving power setting of 60,000 FWHM at

m/z 200 and scan range of *m/z* 100–1000 with normalized AGC target 50% (2e5) and maximum injection time 118 ms. Then the fragmentation spectra of the 6 most intense ions, in positive and negative polarities separately, were recorded with resolution settings of 15,000, AGC target 1.25e4 (25%) and maximum injection time 132 ms with isolation window *m/z* 1.2 and dynamic exclusion of 6s. The HESI source was utilized with settings of capillary voltage 2.5 kV and 3.2 kV in negative and positive ionization mode, respectively. Sheath-, aux- and sweep-gasses were set to 50, 5 and 0 respectively. Capillary temperature was set to 275°C and probe heater was kept at 300°C. The RF lens were set to 55%.

Supplementary Information S5
LC-HRMS data processing

In order to extract features (i.e., chromatographic peaks) from LC-HRMS/MS measurements of standards and plant material samples and to obtain their MS/MS spectra, MZmine (version 4.5.20, https://github.com/mzmine/mzmine/releases/tag/v4.5.20) was utilized. Different workflows for the analytical setups were constructed using the MZwizard of MZmine. The workflow files are available in the folder 'MZmine batch processing files' on Zenodo (https://dx.doi.org/10.5281/zenodo.16322488).

Briefly summarized, the MZmine workflows searched for chromatographic peaks using the LocalMinimumResolver feature detection module. Then, all chromatographic peaks with a certain full width at half maximum (FWHM) were selected. This FWHM was selected to fit most of the chromatographic peaks and was manually optimized. Furthermore, all features without at least one MS/MS spectrum were discarded. The JoinAligner module was used to integrate chromatographic peaks from different samples. Finally, all detected features were exported to a SIRIUS compatible mgf file. Separate entries in these MGF files were generated for different fragmentation methods and collision energy setups. The samples obtained in positive and negative modes were processed separately.

All such generated MGF files are available on Zenodo (https://dx.doi.org/10.5281/zenodo.16322488).

Supplementary Information S6
Comparison of training and test sets

Comparison of training and test sets, as well as compounds assigned to the 'relevant' and 'other' class.

Overlap based on chemical formula (left) and canonicalized SMILES code (right). While there is no overlap in respect to prenylated flavonoids between the train and test dataset, there is a minor overlap in other compounds.

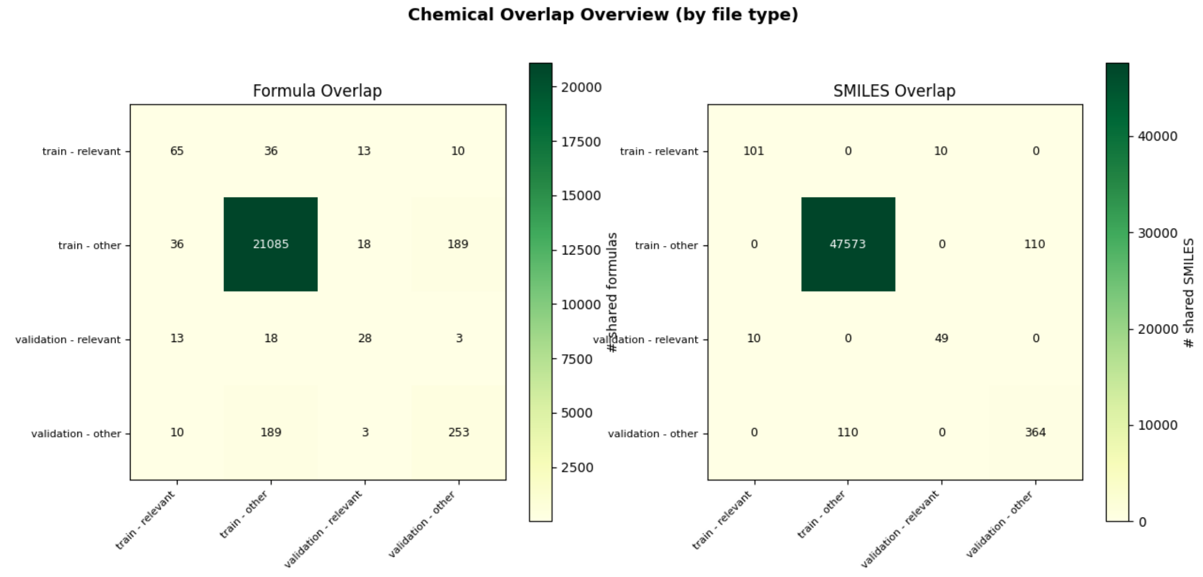

Dimensionality reduction plots calculated from the MS2DeepScore embeddings. Left: Principal Component Analysis, Right: UMAP embedding. In both plots the 'other' class spans a large part of the space (orange dots in the background), while the compounds assigned to the class 'relevant' (i.e., prenylated flavonoids) are located in a small subsection of the entire space (blue and turquoise dots). However, the two dimensionality reduction methods do not completely separate the prenylated flavonoids from all other compounds.

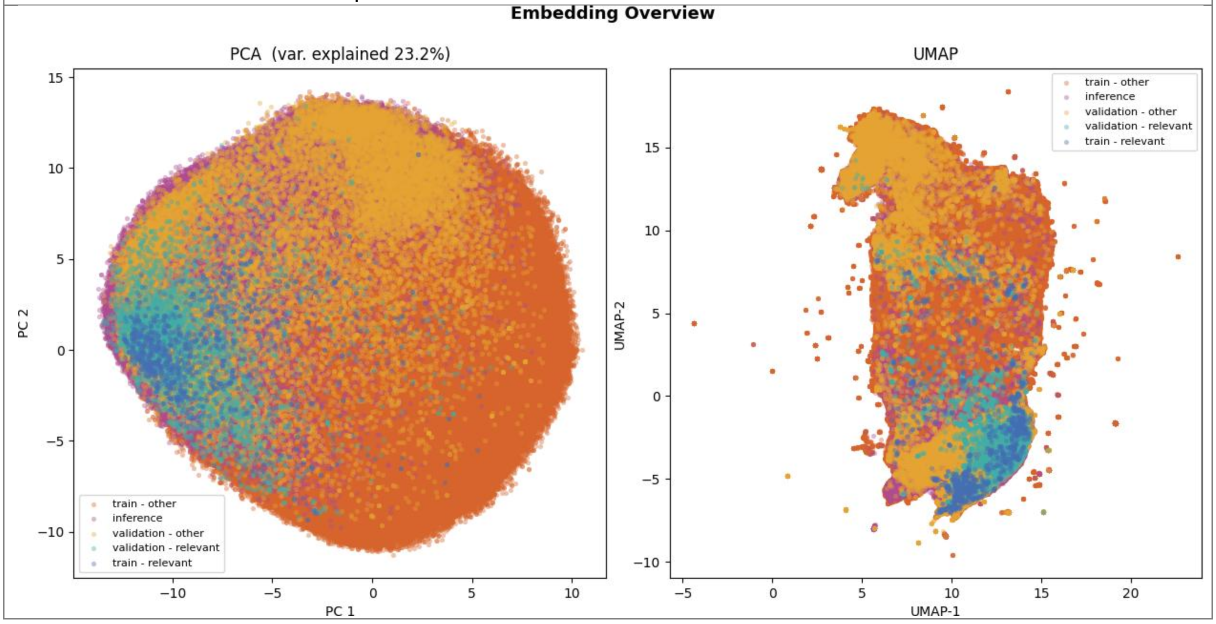

Dimensionality reduction plots calculated from the Morgan fingerprints obtained for train and test compounds from the MSnLib database. Left: Principal Component Analysis, Right: Heatmap plot of 904 randomly selected features. Similar to the PCA plot calculated from the MS2DeepScore embeddings, the PCA scores plot is incapable of completely separating the prenylated flavonoids from all other compounds, however, the prenylated flavonoids are located closely together in a certain subspace of the plot.

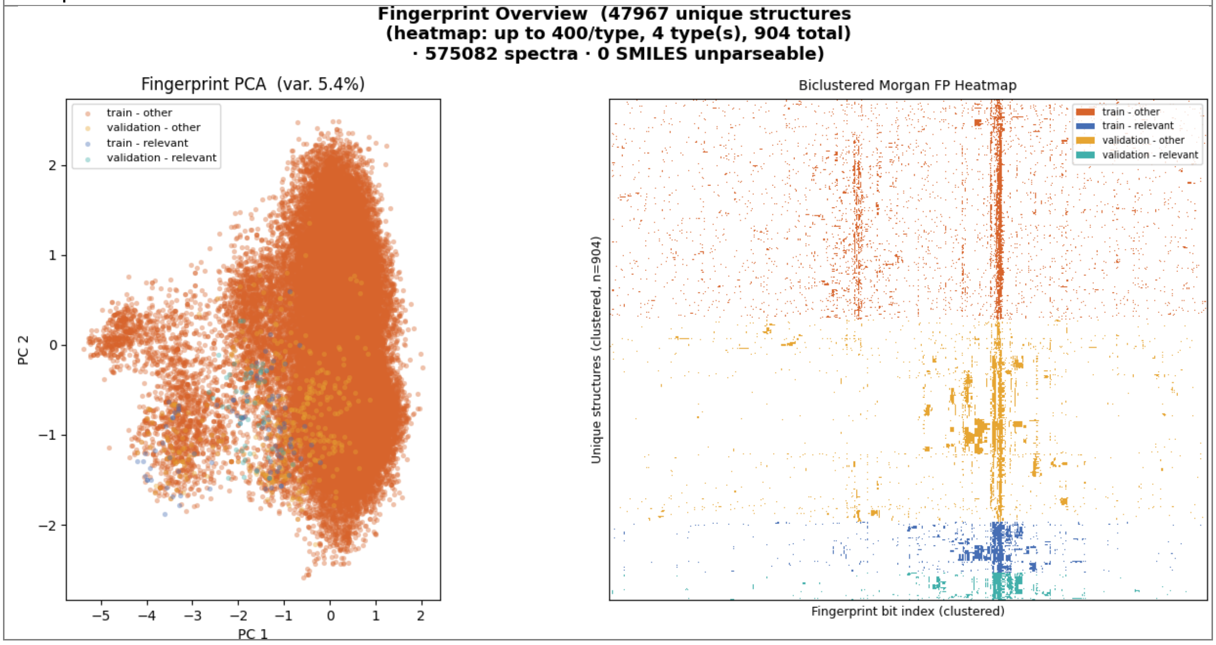

Supplementary Information S7
Data processing parameters of SIRIUS

Command line parameters used to calculate annotations with SIRIUS and CANOPUS. Fallback ions are defined separately for the positive and negative ionization modes.

```
$FALLBACKIONSPOS = "--AdductSettings.fallback=[[M+H]+,[M+Na]+,[M+K]+,[M-H]-]"
$FALLBACKIONSNEG = "--AdductSettings.fallback=[[M-H]-]"

sirius-6.2.2-win-x64\sirius.bat
  --input "$WDIR\$OUTDIR\${Task}__sirius.mgf"
  --output "$WDIR\$OUTDIR\${Task}__sirius.sirius"

config
  --IsotopeSettings.filter=true
  --CandidateFormulas=,
  "--FormulaSettings.enforced=H,C,N,O,P"
  --Timeout.secondsPerInstance=0
  --AlgorithmProfile=orbitrap
  --AdductSettings.ignoreDetectedAdducts=false
  --AdductSettings.prioritizeInputFileAdducts=true
  --UseHeuristic.useHeuristicAboveMz=300
  --IsotopeMs2Settings=IGNORE
  --MS2MassDeviation.allowedMassDeviation=5.0ppm
  --FormulaSearchSettings.performDeNovoBelowMz=400.0
  --FormulaSearchSettings.applyFormulaConstraintsToDatabaseCandidates=false
  --EnforceElGordoFormula=true
  --NumberOfCandidatesPerIonization=1
  "$FALLBACKIONS"
  --FormulaSearchSettings.performBottomUpAboveMz=0
  --FormulaSearchSettings.applyFormulaConstraintsToBottomUp=false
  --UseHeuristic.useOnlyHeuristicAboveMz=650
  --FormulaSearchDB=,
 --Timeout.secondsPerTree=0
  --AdductSettings.enforced=,
  "--FormulaSettings.detectable=B,S,Cl,Se,Br"
  --NumberOfCandidates=10
  --FormulaResultThreshold=true
  --ExpansiveSearchConfidenceMode.confidenceScoreSimilarityMode=APPROXIMATE
  "--StructureSearchDB=CHEBI,COCONUT,GNPS,KEGG,LOTUS,PLANTCYC,SUPERNATURAL"
  --RecomputeResults=false


spectra-search formulas fingerprints classes structures summaries
  --top-k-summary=15
```

Supplementary Information S8 – Comparison of Training Dataset Sizes

To estimate the number of MS/MS spectra necessary to achieve high classification performance, the data from public repository training (Section 3.2 in main manuscript) was reused. To this end, the number of 'relevant' spectra was reduced randomly in 10% steps (starting from 1,778 spectra, down to 178 only), and for each reduction 5 iterations were calculated. Additionally, all available in-house MS/MS spectra were used as a validation dataset to estimate the classification performance. The number of correctly or incorrectly classified spectra per validation dataset was similar across the random iterations per reduction (average of standard deviation 4%, max. of standard deviation 16%). Comparable performance values (on average 62% of the MS/MS spectra of the 'relevant' class were correctly predicted) were observed for the first 5 steps of the training reduction (i.e., 100% down to 50% of the MS/MS spectra used as 'relevant'), indicating that successfully training of the classifier needs at least some 1,000 MS/MS spectra of the 'relevant' class to differentiate them correctly from MS/MS spectra of the class 'other'. The results are illustrated in Figure SIF8 in Supplementary Information SI8.

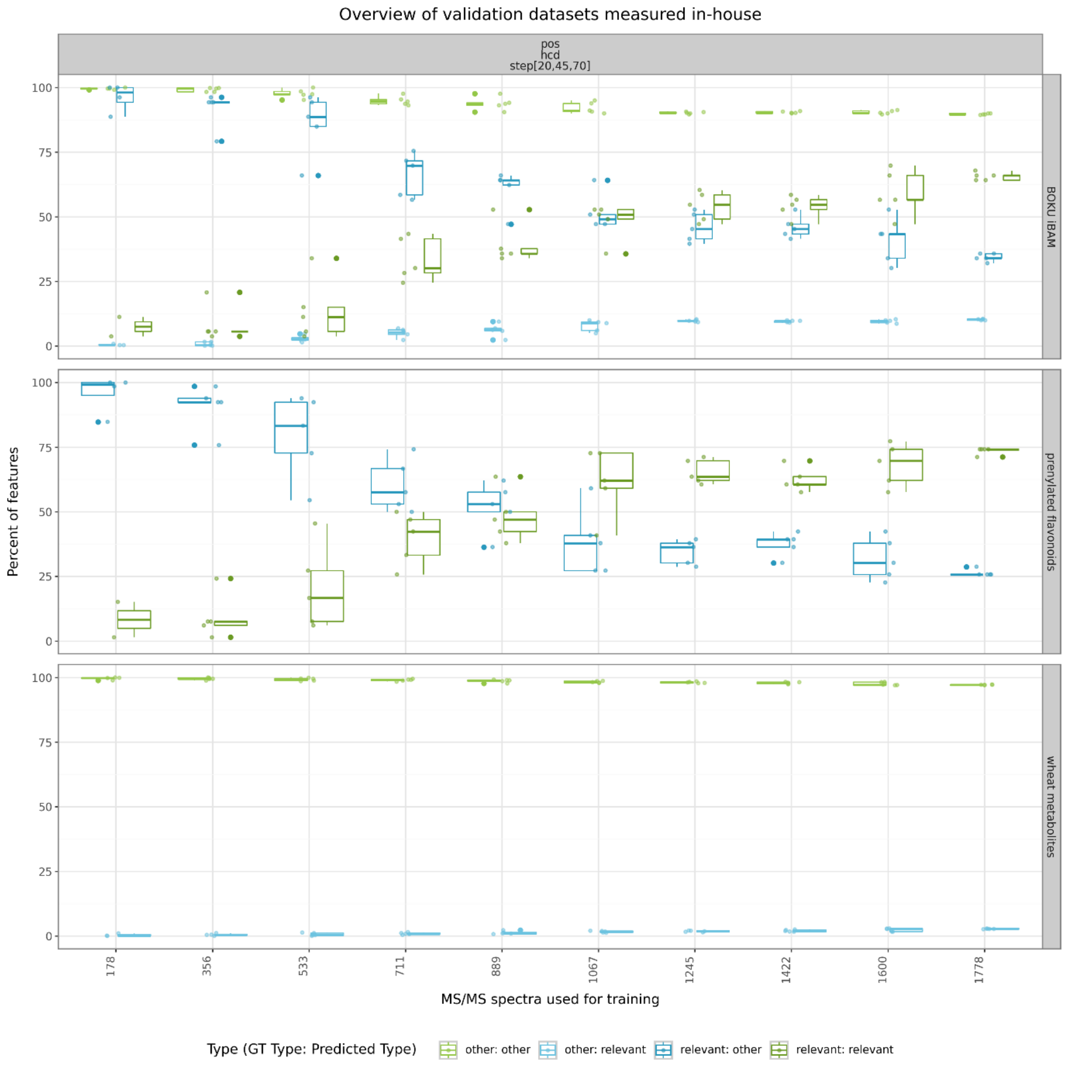

SIF8 | Overview of different number of training instances in the relevant group (x-axis) versus the relative amount of correct or incorrect prediction results. Colors indicate different correctly (green) or incorrectly (blue) predicted classes. For higher numbers of reference spectra (i.e., row of "prenylated flavonoids"), the prediction result is stable with random fluctuations. However, below 1067 reference spectra of "train – relevant" the performance degrades quickly below 50% and with fewer than 533 spectra of "train – relevant" only 25% of the validation dataset spectra are correctly predicted.
Abbreviations: pos: positive ionization mode; hcd: Higher-energy collisional dissociation; step[20, 45, 70]: stepped collision energies of 20, 45, and 70 eV; BOKU iBAM: all MS/MS spectra of the BOKU iBAM in-house library were used for validation; prenylated flavonoids: only the MS/MS spectra of the prenylated flavonoid compounds formed this validation dataset; wheat metabolites: only the MS/MS spectra of the wheat extract formed this validation dataset.
