# Supplementary material for: AnnoMe: user-defined classification of HR-MS/MS spectra for natural product discovery
Source: Bioinform Adv. 2026 May 21;6(1):vbag111. doi: 10.1093/bioadv/vbag111 (PMC13192349; doi:10.1093/bioadv/vbag111)
Supplement: vbag111_Supplementary_Data [file vbag111_supplementary_data.zip › SupplementaryTables.pdf]

AnnoMe: User-defined classification of HR-MS/MS spectra for natural product discovery

Christoph Bueschl[1*], Tomas Rypar[1,2], Lenka Molcanova[3], Juraj Markus[3], Bernhard Seidl[4], Maria Doppler[4], David Ruso[4], Christina Maisl[1], Karel Smejkal[3], Rainer Schuhmacher[1]

[1]BOKU University, Institute of Bioanalytics and Agro-Metabolomics (iBAM), Department of Agricultural Sciences, Vienna, Austria

[2]Mendel University in Brno, Department of Chemistry and Biochemistry, Brno, Czech Republic

[3]Department of Natural Drugs, Masaryk University, Brno, Czech Republic

[4]BOKU University, Core Facility Bioactive Molecules: Screening and Analysis, Tulln, Austria

*To whom correspondence should be addressed.

Supplementary Tables

Contents

ST1 - Metrics for Classification of (Iso-)Flavonoid prediction trained on Public Repository Data

ST2 - Metrics for Classification of Prenylated (Iso-)Flavonoid prediction trained on Public Repository Data

ST3 - Metric for Classification of Prenylated (Iso-)Flavonoid prediction trained on In-House Data

ST4 - Results for Inference on the In-House trained Classifier

Supplementary Table ST1 | Overview of validation dataset for the classification of flavonoids using only the MSnLib public repository as training source.
Numbers are calculated from the Jupyter notebook AnnoME/demo/Classification_FlavonoidsCompounds_publicDBs.ipynb, generated file demo/output/IsoFlavonoids_PublicDBs/summary.xlsx, adapted and extedned with average values.

Split	Subset	n_total	n_relevant	n_other	TP	FN	TN	FP	TP_pct_n_relevant	FN_pct_of_n_relevant	TN_pct_of_n_other	FP_pct_of_n_other	accuracy	balanced_accuracy	F1	AUROC	AUPRC
Train	neg_20.0	19,438	1,212	18,226	792	420	17,922	304	65.3%	34.7%	98.3%	1.7%	0.9628	0.8184	0.6863	0.8419	0.5256
	neg_30.0	7,873	469	7,404	350	119	7,331	73	74.6%	25.4%	99.0%	1.0%	0.9756	0.8682	0.7848	0.8973	0.6779
	neg_60.0	20,130	1,321	18,809	1,043	278	18,512	297	79.0%	21.0%	98.4%	1.6%	0.9714	0.8869	0.7839	0.9026	0.6511
	neg_step[20,45,70]	3,642	452	3,190	376	76	3,137	53	83.2%	16.8%	98.3%	1.7%	0.9646	0.9076	0.8536	0.9416	0.802
	pos_20.0	20,610	1,194	19,416	811	383	19,144	272	67.9%	32.1%	98.6%	1.4%	0.9682	0.8326	0.7123	0.8514	0.5537
	pos_30.0	9,004	336	8,668	244	92	8,621	47	72.6%	27.4%	99.5%	0.5%	0.9846	0.8604	0.7783	0.8793	0.6494
	pos_60.0	20,893	1,233	19,660	975	258	19,455	205	79.1%	20.9%	99.0%	1.0%	0.9778	0.8902	0.8081	0.8984	0.6786
	pos_step[20,45,70]	4,829	271	4,558	199	72	4,530	28	73.4%	26.6%	99.4%	0.6%	0.9793	0.8641	0.7992	0.9166	0.7435
														**average**	**sd**	**min**	**max**
														**0.866**	**2.8%**	**0.818**	**0.908**
Test	neg_20.0	329	177	152	113	64	144	8	63.8%	36.2%	94.7%	5.3%	0.7812	0.7929	0.7584	0.7956	0.793
	neg_30.0	325	177	148	115	62	140	8	65.0%	35.0%	94.6%	5.4%	0.7846	0.7978	0.7667	0.8154	0.8137
	neg_60.0	306	177	129	98	79	124	5	55.4%	44.6%	96.1%	3.9%	0.7255	0.7575	0.7	0.7801	0.8037
	neg_step[20,45,70]	317	177	140	138	39	128	12	78.0%	22.0%	91.4%	8.6%	0.8391	0.847	0.844	0.8828	0.8714
	pos_20.0	525	229	296	169	60	291	5	73.8%	26.2%	98.3%	1.7%	0.8762	0.8605	0.8387	0.8821	0.8564
	pos_30.0	516	223	293	142	81	281	12	63.7%	36.3%	95.9%	4.1%	0.8198	0.7979	0.7533	0.8322	0.7812
	pos_60.0	496	220	276	159	61	259	17	72.3%	27.7%	93.8%	6.2%	0.8427	0.8306	0.803	0.8502	0.7982
	pos_step[20,45,70]	514	227	287	185	42	268	19	81.5%	18.5%	93.4%	6.6%	0.8813	0.8744	0.8585	0.9051	0.853
														**average**	**sd**	**min**	**max**
														**0.820**	**3.7%**	**0.758**	**0.874**

Supplementary Table ST2 | Overview of predictions for the validation set of prenylated flavonoids (from reference standards) and wheat metabolites.
Compounds of the class 'relevant' are prenylated flavonoids, while compounds of the class 'other' can be anything but the former types.
Calculated via GUI (uv run annome_classificationgui), loaded project configuration AnnoMe/demo/GUI_PrenylatedCompounds_ClassificationProject.json, results compiled from AnnoMe/output/classification_results_PrenylatedFlavonoids/summary.xlsx

Split	Subset	n_total	n_relevant	n_other	TP	FN	TN	FP	TP_pct_n_relevant	FN_pct_of_n_relevant	TN_pct_of_n_other	FP_pct_of_n_other	accuracy	balanced_accuracy	F1	AUROC	AUPRC
Train	neg_step[20,45,70]	10,166	71	10,095	71	0	9,841	254	100.0%	0.0%	97.5%	2.5%	0.975	0.9874	0.3586	1	0.9958
	pos_step[20,45,70]	20,537	28	20,509	28	0	20,181	328	100.0%	0.0%	98.4%	1.6%	0.984	0.992	0.1458	1	1
														**average**	**min**	**max**	
														**0.990**	**0.987**	**0.992**	
Test	neg_step[20,45,70]	3,269	115	3,154	88	27	2,634	520	76.5%	23.5%	83.5%	16.5%	0.8327	0.8002	0.2434	0.8633	0.4023
	pos_step[20,45,70]	4,043	115	3,928	89	26	3,721	207	77.4%	22.6%	94.7%	5.3%	0.9424	0.8606	0.4331	0.9472	0.4307
														**average**	**min**	**max**	
														**0.830**	**0.800**	**0.861**	
	Non-prenylated (Iso-)Flavonoids_neg_step[20,45,70]	130		130			75	55			57.7%	42.3%					
	Non-prenylated (Iso-)Flavonoids_pos_step[20,45,70]	180		180			120	60			66.7%	33.3%					

Supplementary Table ST4 | Results for cross validation (10 fold) on the in-house training dataset.

Numbers are calculated from the Jupyter notebook AnnoME/demo/Classification_PrenylatedCompounds_BOKUDB.ipynb, generated file demo/output/PrenylatedCompounds_BOKUDB/summary.xlsx, adapted and extended with average values.

Split	Subset	n_total	n_relevant	n_other	TP	FN	TN	FP	TP_pct_n_relevant	FN_pct_of_n_relevant	TN_pct_of_n_other	FP_pct_of_n_other	accuracy	balanced_accuracy	F1	AUROC	AUPRC
Train	neg_20.0	396	122	274	107	15	274	0	87.7%	12.3%	100.0%	0.0%	**0.9621**	0.9385	0.9345	1	1
	neg_30.0	392	122	270	102	20	270	0	83.6%	16.4%	100.0%	0.0%	**0.949**	0.918	0.9107	1	1
	neg_40.0	1,615	122	1,493	102	20	1,484	9	83.6%	16.4%	99.4%	0.6%	**0.982**	0.915	0.8755	0.9536	0.8479
	neg_step[20,45,70]	1,633	121	1,512	103	18	1,508	4	85.1%	14.9%	99.7%	0.3%	**0.9865**	0.9243	0.9035	0.9567	0.8955
	pos_20.0	525	55	470	31	24	470	0	56.4%	43.6%	100.0%	0.0%	**0.9543**	0.7818	0.7209	1	1
	pos_30.0	516	55	461	39	16	461	0	70.9%	29.1%	100.0%	0.0%	**0.969**	0.8545	0.8298	1	1
	pos_40.0	510	55	455	36	19	455	0	65.5%	34.5%	100.0%	0.0%	**0.9627**	0.8273	0.7912	1	1
	pos_step[20,45,70]	514	55	459	40	15	459	0	72.7%	27.3%	100.0%	0.0%	**0.9708**	0.8636	0.8421	1	1
														**average**	**sd**	**min**	**max**
														**0.878**	**5.2%**	**0.782**	**0.939**

Supplementary Table ST5 | Results for inference on the in-house trained classifier for 'relevant' (prenylated flavonoids) and 'other'

**Source**	**File**	**Subset**	**annotated_as**	**n_features**	**percent_features**
Glycyrrhizza uralensis	n_GU	neg_step[20,45,70]	other	677	74.6%
			relevant	231	25.4%
		pos_step[20,45,70]	other	1,681	84.6%
			relevant	305	15.4%
Paulownia tomentosa	n_PT22	neg_step[20,45,70]	other	1,351	65.3%
			relevant	718	34.7%
		pos_step[20,45,70]	other	4,242	80.8%
			relevant	1,005	19.2%
	n_PT24	neg_step[20,45,70]	other	1,336	68.6%
			relevant	612	31.4%
		pos_step[20,45,70]	other	4,303	81.3%
			relevant	989	18.7%
	PT22CH	pos_step[20,45,70]	other	827	82.9%
			relevant	171	17.1%
